# Visualization of the impatiens downy mildew pathogen using fluorescence in situ hybridization (FISH)

**DOI:** 10.1186/s13007-018-0362-z

**Published:** 2018-10-24

**Authors:** Catalina Salgado-Salazar, Gary R. Bauchan, Emma C. Wallace, Jo Anne Crouch

**Affiliations:** 1 Agriculture Research Service (ARS), Mycology and Nematology Genetic Diversity and Biology Laboratory, U.S. Department of Agriculture, 10300 Baltimore Avenue, Beltsville, MD 20705 USA; 2 ARS Research Participation Program, Oak Ridge Institute for Science and Education, MC-100-44, P.O. Box 117, Oak Ridge, TN 37831 USA; 3 Agriculture Research Service, Electron and Confocal Microscopy Unit, U.S. Department of Agriculture, 10300 Baltimore Avenue, Beltsville, MD 20705 USA; 4 Present Address: Department of Plant Pathology and Environmental Microbiology, The Pennsylvania State University, 120 Buckhout Lab, University Park, PA 16802 USA

**Keywords:** Oomycota, Peronosporales, Oospores, Downy mildew, Natural environment

## Abstract

**Background:**

*Plasmopara obducens* is the biotrophic oomycete responsible for impatiens downy mildew, a destructive disease of *Impatiens* that causes high crop loss. Currently, there are no available methods for the microscopic detection of *P. obducens* from leaves of impatiens, which may be contributing to the spread of the disease. Fluorescence in situ hybridization (FISH) is a sensitive and robust method that uses sequence-specific, fluorescence-labeled oligonucleotide probes to detect target organisms from the environment. To study this important pathogen, we developed and standardized a FISH technique for the visualization of *P. obducens* from *Impatiens walleriana* tissues using a species-specific 24-mer oligonucleotide probe designed to target a region of the rRNA internal transcribed spacer 2 (ITS2).

**Results:**

Since *P. obducens* cannot be propagated in vitro, we developed a custom *E. coli* expression vector that transcribes the *P. obducens* rRNA-ITS target sequence (clone-FISH) for use as a control and to optimize hybridization conditions. The FISH assay could detect *P. obducens* sporangiophores, sporangia and oospores, and hyphae from naturally infected *I. walleriana* leaves and stems. Cross-reactivity was not observed from plant tissue, and the assay did not react when applied to *E. coli* with self-ligated plasmids and non-target oomycete species.

**Conclusions:**

This FISH protocol may provide a valuable tool for the study of this disease and could potentially be used to improve early monitoring of *P. obducens*, substantially reducing the persistence and spread of this destructive plant pathogen.

## Background

Impatiens downy mildew caused by the obligate biotroph *Plasmopara obducens* (*Oomycota*, *Peronosporales*) is one of the most devastating diseases of the ornamental bedding plant *Impatiens walleriana*, and also may affect interspecific hybrids and related wild species of *Impatiens* (impatiens; [1–3]). Symptoms of impatiens downy mildew (IDM) include leaf yellowing, stunted growth, leaf drop and stem collapse [4]. As the disease progresses, a white downy-like growth on the underside of leaves can be observed, corresponding with the emergence of asexual fruiting structures (sporangiophores and zoospore-bearing sporangia) from the leaf stomata [5]. Oospores, the pathogen’s primary survival structure, are formed in stems of plants that have recently died, and may serve as a primary inoculum source for subsequent growing seasons in landscape settings [6]. *Plasmopara obducens* sporangia are spread by wind currents or by water from rain or irrigation, and under cool and moist conditions, the disease spreads rapidly [6].

Epidemic outbreaks of IDM on *I. walleriana* were first reported in the early 2000s in the UK, Europe and Australia [7–10]. In the United States, the first epidemic outbreaks that reached landscape settings started during the 2011 and 2012 growing seasons [1, 2, 11–14]. Currently, IDM is reported throughout the continental US and the Hawaiian Islands, and continues to limit the health and production of this economically important crop, worth $120 million as of 2014 (USDA-National Agricultural Statistics Service Census of Agriculture 2015 report, https://www.agcensus.usda.gov, [3]). Diagnosis of IDM relies on the presence of typical symptoms that can include leaf yellowing and stunted growth, and at later stages of the disease, is aided by the visible signs of the pathogen’s vegetative and fruiting bodies on the undersurface of infected leaves. However by the time that disease symptoms and visual signs of the pathogen appear, the disease is incurable: plants cannot be treated and losses cannot be avoided. Additionally, plants may be infectious long before detectable symptoms appear. Early detection methods aimed at identifying the infection status of apparently uninfected individuals is key for efficient use of disease control resources [15].

Fluorescence in situ hybridization (FISH) has been widely used as a cultivation-independent tool for direct detection, identification and quantification of microorganisms [16]. The cultivation-independent characteristic of this technique is also particularly important for microorganisms such as obligate biotrophs, as it allows for the direct study of plant pathogens in their natural environment [17, 18]. Although updated techniques, applications and protocol improvements are now available for FISH, the technique is based around four core steps: (1) specimen fixation and immobilization; (2) permeabilization to increase accessibility of an organism specific-nucleic acid probe to the target; (3) hybridization of the probe; (4) washing to remove unbound probe; and (4) documentation by microscopy or flow cytometry [16, 19, 20]. Typical oligonucleotide probes used for FISH range between 15 and 30 base pairs in length and are labeled with one or more fluorescent dyes [20]. Most FISH applications target ribosomal RNA (rRNA), as these molecules are highly abundant and stable within cells, and possess both variable and highly conserved sequence domains [19]. Single copy genes can also be detected using FISH when coupled with signal amplification techniques such as catalyzed reporter deposition—CARD-FISH [21]. Even though FISH assays using oligonucleotide probes targeting rRNA were first introduced almost 30 years ago [22], only few studies have applied this technique for the visualization of oomycete plant pathogens such as *Phytophthora agathidicida* and *P. cinnamomi* [23–25]. Non-specific fluorescent staining techniques have been used to visualize infection structures and plant cellular growth and response to the grape downy mildew pathogen *Plasmopara viticola* [26–28], and to visualize the *in planta* development of pathogens such as *Peronospora sparsa*, *Pe. tabacina, Pseudoperonospora cubensis*, and *Ps. humuli*, causing rose, tobacco, cucurbit, and hops downy mildew, respectively [28, 29]. However, to date, FISH assays have not been developed for species-specific visualization of oomycetes that cause downy mildew diseases.

The aim of the current study is to develop a detailed protocol for species-specific FISH visualization of the impatiens downy mildew pathogen. For this, a specific oligonucleotide probe targeting the rRNA internal transcribed spacer (ITS) region of *P. obducens* was developed and tested using the clone-FISH approach and then validated with *P. obducens* mycelia, sporangiophores, sporangia and oospores harvested from *I. walleriana,* as well as *I. walleriana* leaves and stems showing symptoms of downy mildew. The development of a FISH probe and hybridization assay allowed the microscopic visualization of *P. obducens* within *I. walleriana* plant tissues, easily distinguishable from plant cells. This technique could be a useful tool for pathogen detection on non-symptomatic *I. walleriana*, as well as a tool to study *P. obducens* life cycle including key cellular events such as host penetration and colonization.

## Materials and methods

### Probe design

The oligonucleotide probe used in this study (rRNA_ITS_Pob) was designed to specifically target *P. obducens*. This was achieved by comparing the *P. obducens* rRNA ITS 1 and 2 regions (including the 5.8S rRNA gene) [1] with publicly available rRNA ITS sequences retrieved from NCBI GenBank of closely related oomycetes in the *Peronosporaceae* family and oomycete plant pathogens commonly found inhabiting soil. The rRNA_ITS_Pob probe (5′-ACCAAACTGGTCGCCGACTTGTTA-3′) has a melting temperature of 59 °C, G–C content 45.8%, phosphorothioate bonds (to be resistant to nuclease degradation), and was synthesized commercially (IDT, Coralville, IA, USA). Preliminary assessments of autoflorescence emitted by *I. walleriana* leaves, stems and flowers revealed broad emissions in the green spectra (data not shown), which precluded the use of green spectra fluorophores such as GFP. Two versions of the probe within the blue and red emission spectra were synthesized: one probe with an AlexaFluor350 fluorophore (Invitrogen, Rockville, MD) at the 5′ end (excitation = 346 nm, emission = 442 nm [blue]) and a second probe with an AlexaFluor594 fluorophore at the 5′ end (excitation = 590 nm, emission = 617 nm [red]). The spectral properties of these two fluorophores (excitation and emission maximum wavelengths, brightness and photostability) made them the most appropriate for this application.

### Preparation of control materials: clone-FISH cells and non-target oomycetes

In the absence of pure cultures of *P. obducens* that could be used as controls to evaluate the rRNA_ITS_Pob probe before its application on impatiens samples, a clone-FISH protocol was used to develop *E. coli* cells expressing the *P. obducens* target sequence, following the general method of Schramm et al. [30]. DNA from *P. obducens* sample DE14.2.9 [3] was extracted using the OmniPrep kit (G-Biosciences, St. Louis, MO, USA) and purified using the Zymo DNA Clean and Concentrator kit (Zymo Research, Irvine, CA, USA). The ~ 1 kb *P. obducens* target rRNA ITS 1 and 2 regions (including the 5.8S rRNA gene) was amplified by PCR using the DC6-PL-OB-3′-699 primer set (DC6: 5′-GAGGGACTTTTGGGTAATCA-3′; PL-OB-3′-699: 5′-TTAGAAGACCAAGCAACTCG-3′) with cycling conditions as follows: initial denaturation at 95 °C for 5 min, followed by 35 cycles of 95 °C for 30 s, 51 °C for 45 s and 72 °C for 45 s, and final extension at 72 °C for 10 min. The PCR product was purified using the Wizard SV Gel and PCR Clean-up System (Promega, Madison, WI, USA), ligated into the pCR 2.1-TOPO vector and inserted into *Escherichia coli* TOP10F’ competent cells (Invitrogen, Carlsband, CA, USA). Forty-eight clones were randomly picked and the insertion and direction of ligated PCR product was checked by Sanger sequencing using the M13 forward–reverse primer set included in the TOPO^®^ TA Cloning Kit (Invitrogen, Carlsband, CA, USA). One *E. coli* colony containing plasmid constructs with the insert in the correct orientation was purified using the QIAprep Spin Miniprep Kit (Qiagen, Germantown, MD, USA) and used to transform *E. coli* BL21 Star (DE3) One Shot cells (Invitrogen, Carlsband, CA, USA) containing a genomic copy of IPTG-inducible T7 RNA polymerase to generate sufficient transcript for hybridization purposes. One *E. coli* colony with plasmids containing the insert in the opposite direction and one *E. coli* colony carrying a self-ligated plasmid (without the insert) were used as negative controls and used to transform *E. coli* DE3 cells. Cells were cultivated in fresh LB broth in a shaking incubator at 37 °C until they reached a mid-log phase (OD_600_ of 0.3–0.4). IPTG (isopropyl-β-d-thiogalactopyranoside) in a final concentration of 0.5 mM was added to the cultures and allowed to incubate for 1 h to induce transcription of the insert sequence. Chloramphenicol (170 g L^−1^) was added to the cell culture for 4 h and cells were then fixed overnight in 4% formaldehyde solution at 4 °C. After fixation, clones were pelleted, washed in 1 × PBS (phosphate-buffered saline) buffer twice, and stored in 1 × PBS/absolute ethanol (1:1) at − 20 °C until needed for FISH treatment.

Slide cultures of *Phytophthora infestans*, *Phy. sojae* and *Pythium irregulare* were prepared according to Riddell [31] to test for cross reactivity between the *P. obducens* FISH probe and other oomycetes that commonly reside in agricultural soil. For this, mycelia of *Phy. infestans*, *Phy. sojae* and *Py. irregulare* was collected from 1-week old cultures on rye agar medium supplemented with 2% glucose (*Phy. infestans* and *Phy. sojae*) and potato dextrose agar (PDA, *Py. irregulare*) and inoculated at the edge of agar blocks on glass slides following the method described by Riddell [31]. The samples on the resulting slide cultures were processed immediately.

### Collection and preparation of *Plasmopara obducens* samples for imaging

Mycelia, sporangia and sporangiophores of *P. obducens* were obtained from naturally infected *I. walleriana* plants collected in Montgomery County, MD. Mycelia, sporangia and sporangiophores were collected from the underside of the leaves using an entomological needle and with the aid of a Zeiss dissecting microscope Discovery V20 (Carl Zeiss Microscopy, Thornwood, NY, USA). Oospores were obtained by inoculating surface-disinfested (10% commercial bleach for 60 s) stem segments of healthy *I. walleriana* with a suspension of freshly collected sporangia (1 × 10^5^ spores mL^−1^). Inoculated stems were kept in moist chambers consisting of moist filter paper in a 90 mm Falcon sterile polystyrene petri plates (Becton–Dickinson Labware, Franklin Lakes, NJ, USA) and incubated with a 14 h photoperiod for approximately 1 month at 20 °C to allow the oospores to develop and mature.

### Fluorescence in situ hybridization

In preparation for hybridization, samples of *E. coli*, *Phy. infestans*, *Phy. sojae*, *P. obducens* and *Py. irregulare* were heat fixed onto glass microscope slides by incubating the slide on the surface of a 60 °C hotplate for 15 s. After incubation, a 65 µL capacity Frame-seal^©^ (BIO-RAD Laboratories, Hercules, CA, USA), was placed on the slides around the samples.

Fresh leaves and stems of *I. walleriana* naturally infected with *P. obducens* were prepared for hybridization by fixing overnight in 4% formaldehyde solution at 4 °C. After fixation, the leaves and stems were washed in 1 × PBS buffer twice and tissue cleared using a solution of ethanol 95%:acetic acid:glycerol (75:15:10 v/v). The fixed plant material can be stored in 1 × PBS/absolute ethanol (1:1) at 4 °C for up to 6 months. Hybridization of leaves and stems was performed in sterile polystyrene petri plates (Falcon 35 × 10 mm; Becton–Dickinson Labware, Franklin Lakes, NJ, USA).

Fluorescence in situ hybridization (FISH) of prepared materials was performed following the general protocol of Matthiesen and Hansen [32], which replaces the use of formamide with non-toxic organic solvents, increasing the hybridization rate and reducing the temperature required to perform the denaturation and hybridization steps [32, 33]. Briefly, samples were dehydrated through a graded series of ethanol (2 min 70% ethanol, 2 min 85% ethanol, 2 min 96% ethanol) and air-dried. Dehydrated samples were incubated with hybridization buffer (15% v/v ethylene carbonate, 20% v/v dextran sulfate, 600 mM NaCl, 10 mM citrate buffer, 2 ng L^−1^ fluorescent probe, pH 6.2) at 67 °C for 10 min, followed by incubation at 45 °C for 1 h. 50 µL of hybridization buffer was used on slides and approximately 200 µL was used for incubation of leaf and stem tissue. After hybridization, samples were washed three times: an initial 10 min wash at 65 °C in buffer 1 (0.05 M Tris–HCl, 0.3 M NaCl, 0.1% Tween 20, pH 7.6), followed by two room temperature washes in buffer 2 (0.05 M Tris–HCl, 0.15 M NaCl, 0.1% Tween 20, pH 7.6) for 3 min. Washed samples were dehydrated through a graded series of ethanol (2 min 70% ethanol, 2 min 85% ethanol, 2 min 96% ethanol) and air-dried. Samples mounted on glass microscope slides had their Frame-seal^©^ removed and a glass cover slip was added to the samples with a drop of ProLong^®^ Diamond Antifade Mountant (ThermoFisher, Carlsband, CA, USA) and cured overnight at 4 °C in the dark.

### Microscopy and image acquisition

Fluorescence microscopy was performed with a Zeiss Axio Imager.M2 microscope equipped with an HXP120V fluorescent light source and filter sets 49 (excitation G365, emission BP 445/50) and 64 HE (excitation BP587/25, emission 647/70) (Carl Zeiss Microscopy, Thornwood, NY, USA). Images were acquired in gray scale using an Axiocam 506 Mono digital camera (Carl Zeiss Microscopy) and processed using Zen 2 Pro Software (Carl Zeiss Microscopy). Lower magnification images were obtained using a Zeiss Axio Zoom.V16 steromicroscope system (Carl Zeiss Microscopy). The exposure time for each channel was auto-balanced prior to image acquisition in order to avoid oversaturation of signal or bleaching of the probe. Exposure times obtained for the fluorescent channels using a positive sample resulting from the clone-FISH assay and *P. obducens* mycelia were subsequently used for the evaluation of the probes hybridized against *I. walleriana* infected leaves and negative controls (other oomycetes and *E. coli* colony carrying a self-ligated plasmid). Confocal images of oospores were obtained using a Zeiss LSM710 confocal laser scanning microscopy (CLSM) system (Carl Zeiss Microscopy). CLSM images were generated using a Zeiss Axio Observer inverted microscope with 10 × 0.45 NA and 25 × 0.8 NA Plan-Apochromat objectives. Two lasers were used, 488 nm argon laser and 561 nm diode-pumped solid state laser with a pin hole of 33 μm passing through a MBS 488 or MBS 561 beam splitter filter with limits set between 490 and 530 nm for detection using the 488 nm laser and 565–650 nm for detection using the 561 nm laser. Zeiss Zen 2012 (Carl Zeiss Microscopy) software was used to obtain 20–30 Z-stack images and a maximum intensity projection was used to develop the final 2D image.

## Results and discussion

### Validation of rRNA ITS-targeted oligonucleotide probe

**Fig. 1:**
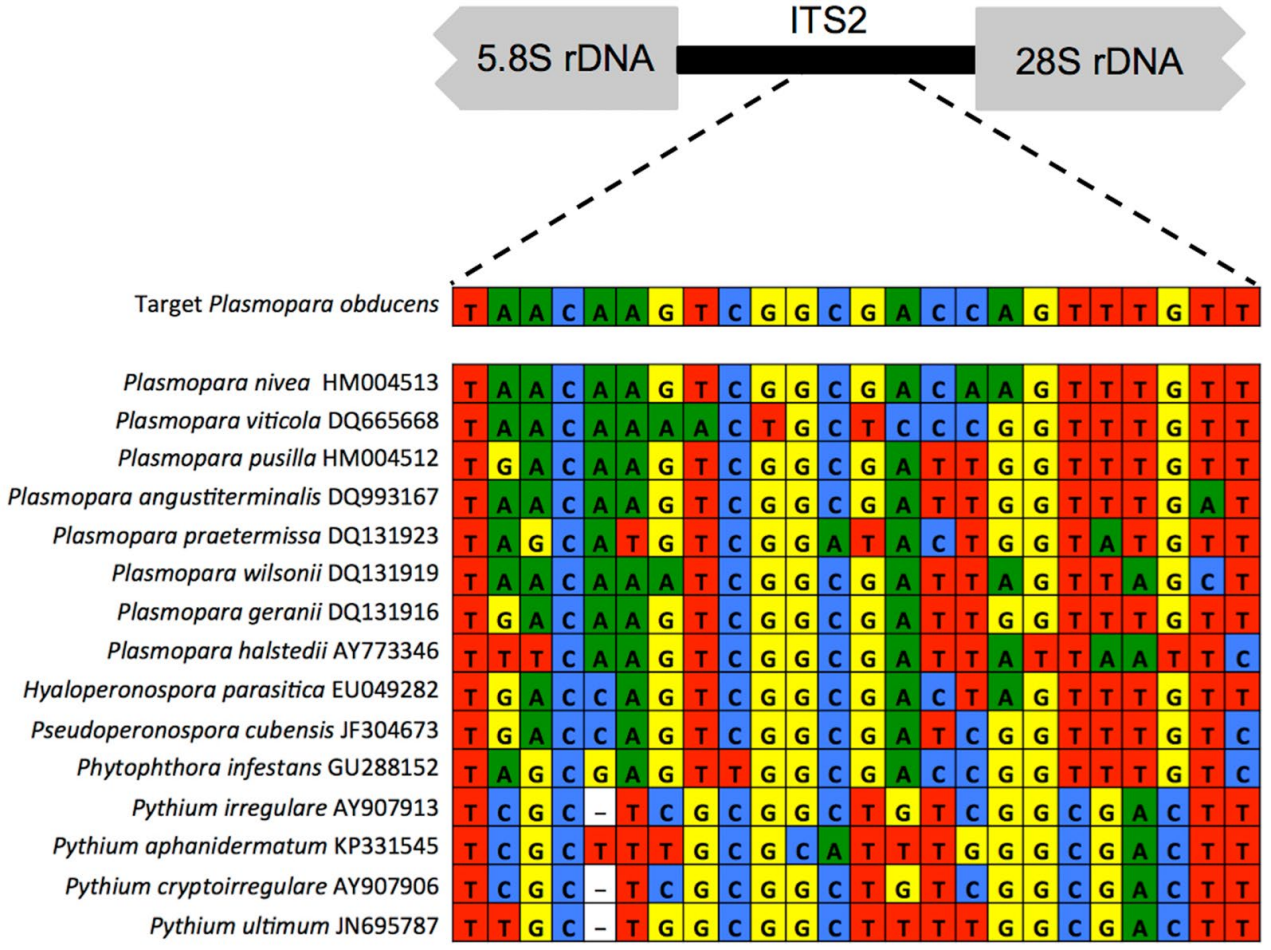
Aligned portion of the rRNA ITS2 target region used to develop the fluorescent in situ hybridization probe used for visualization of *P. obducens*. Probe design was based on the nucleotide differences between *P. obducens* and non-target organisms: other *Plasmopara* species, other downy mildew-causing species in the Peronosporaceae and oomycete plant pathogens commonly found on soil. Sequences from non-target organisms share between 75 and 42% similarity with the *P. obducens* probe

The 24-nucleotide long rRNA_ITS_Pob probe designed in this study was based on the nucleotide divergence between *P. obducens* ITS rRNA region and that of other *Plasmopara* species and Peronosporaceae taxa (Fig. 1). The region used to develop the probe was located within the ITS 2 region and possessed ideal nucleotide composition for use as a probe (data not shown). Additional probe regions in the ITS rRNA regions with enough species-specific nucleotides were not identified. BLASTn searches against NCBI GenBank showed that ITS sequences from two samples of the lettuce downy mildew pathogen *Bremia lactucae* (host = *Hemistepta lyrata*) shared significant similarity with the probe, with 100% identity over 79% of the sequence (e-value = 2.3; accessions DQ235793, DQ235794). Other members of the genus *Plasmopara* shared a maximum of 96% identity over 88% of the probe sequence (e.g. *P. nivea* EF553508, host = *Aegopodium podagraria*; *P. angustiterminalis* DQ993167, host = *Xanthium strumarium*; e-value = 1e − 04). However, given the fact that none of these Peronosporaceae organisms are known to inhabit impatiens, cross-reactivity with these organisms from impatiens samples is extremely unlikely.

**Fig. 2:**
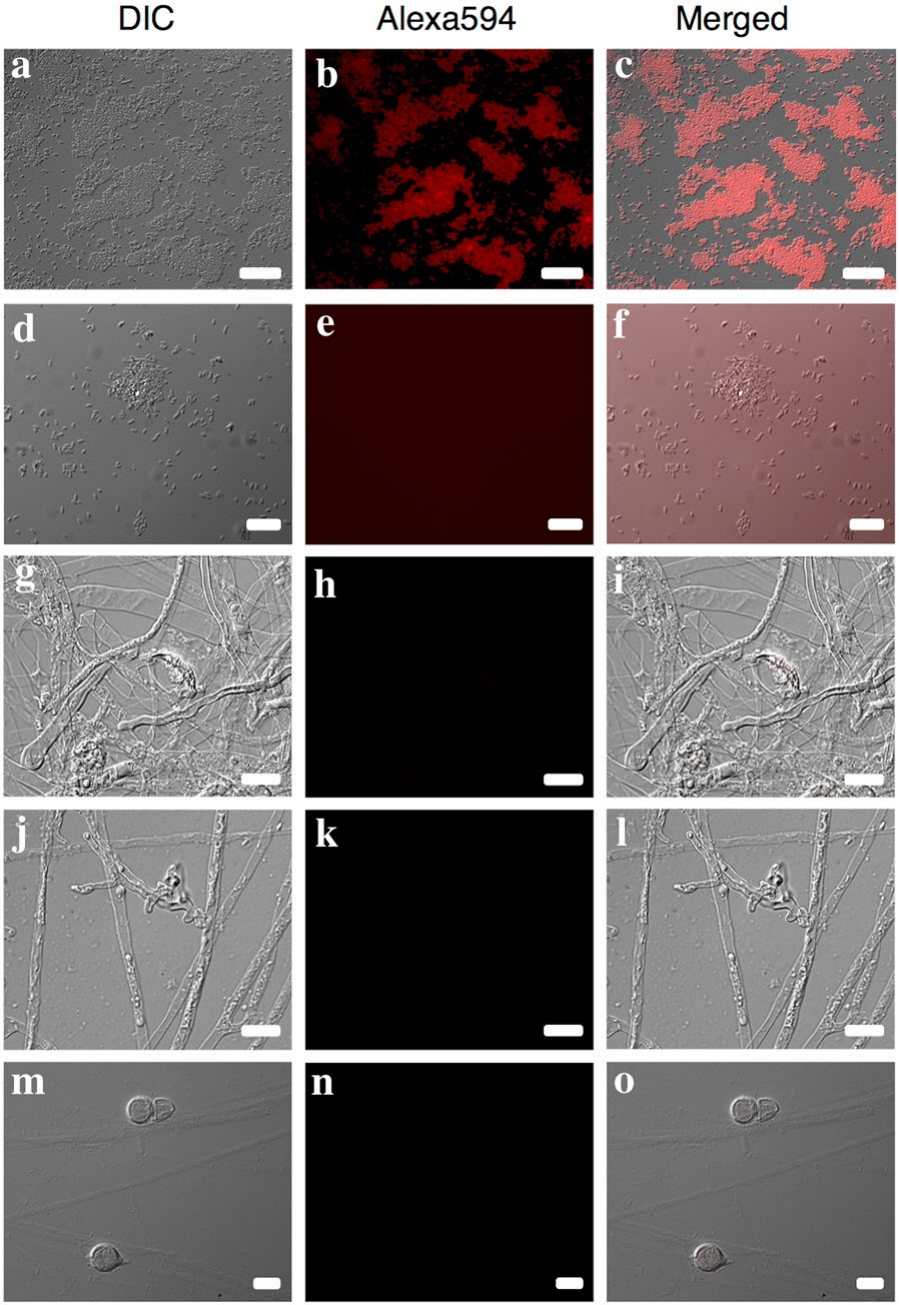
Probe validation using clone-FISH and evaluation of cross-reactivity with non-target oomycetes. **a**–**c** Positive control: *Escherichia coli* DE3 cells expressing the *Plasmopara obducens* target sequence; **d**–**f** negative control: *E. coli* DE3 cells carrying a self-ligated plasmid without the *P. obducens* target sequence insert; **g**–**i** negative controls: *Phytophthora infestans* mycelia and spore; **j**–**l** *Phy. sojae* mycelia; **m**–**o**, *Pythium irregulare* mycelia and spores. Scale bar: **a**–**c**, **g**–**o** = 20 µm; **d**–**f** = 10 µm

Exposure times for images of clone-FISH and *P. obducens* positive samples using the Alexa-350 and Alexa-594-labeled probes varied according to the sample assayed, with clone-FISH cells requiring exposure times an order of magnitude longer (2.6–5.2 s exposure; Fig. 2a–c) than *P. obducens* mycelia and oospores (50–500 ms exposure) or pathogen-infected leaf tissue (5–20 ms exposure). By reusing the fluorescent channel exposure time of positive samples, no fluorescence signal was detected from *E. coli* DE3 strains carrying self-ligated plasmids (Fig. 2d–f), slide preparations of *Phy. infestans*, *Phy. sojae* and *Py. irregulare* (Fig. 2g–o) or from healthy *I. walleriana* leaf tissue (not shown).

FISH assay of *P. obducens* mycelia and *I. walleriana* infected leaves and stems (oospores).

**Fig. 3:**
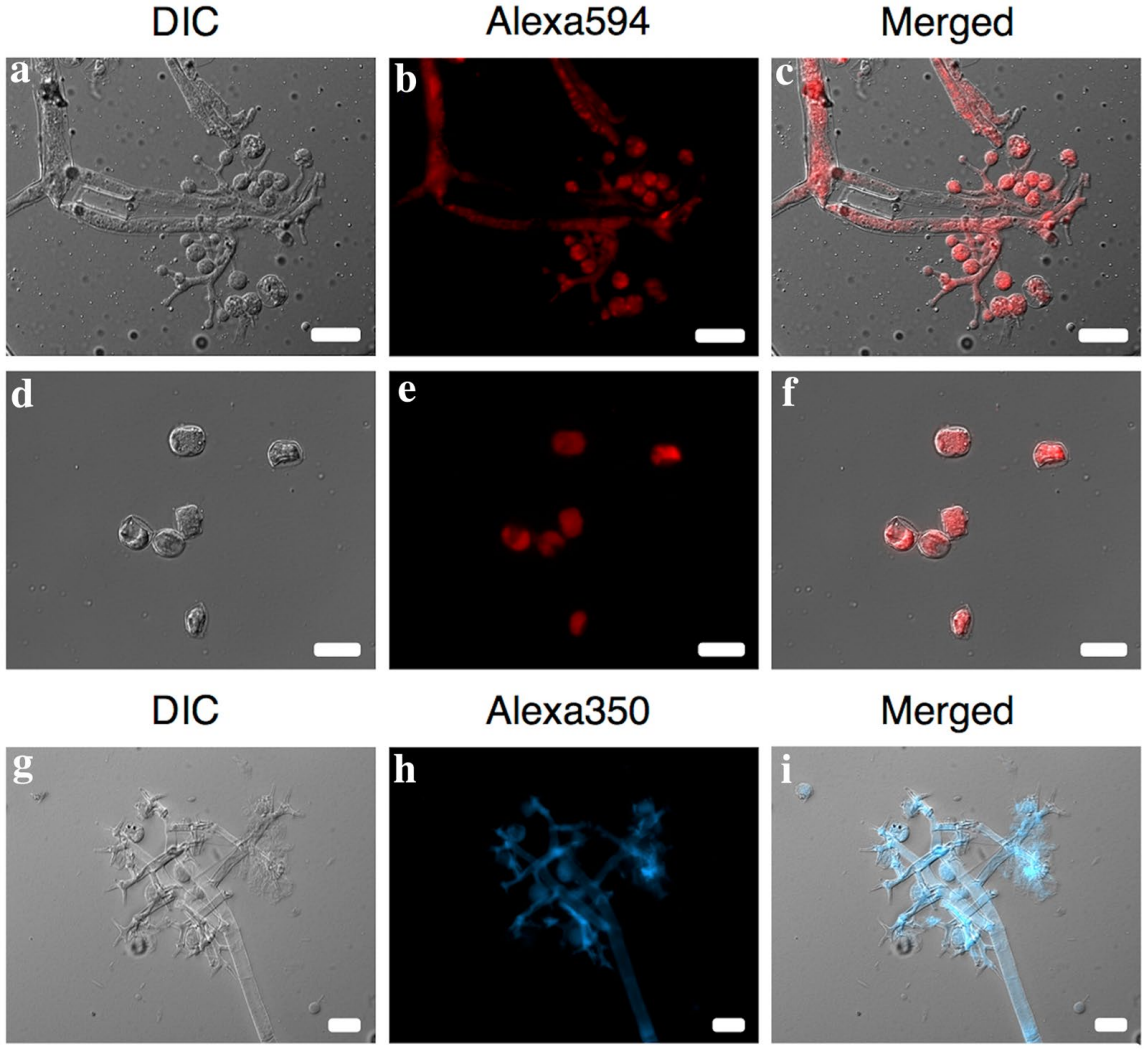
*Plasmopara obducens* sporangiophores and sporangia hybridized to probes labeled with Alexa595 (red, **a**–**f**) or Alexa350 (blue, **g**–**i**) fluorophores. Scale bar = 20 µm

The FISH assay directly applied to *P. obducens* sporangiophores and sporangia fixed onto glass slides showed the rRNA_ITS_Pob probe exhibited strong fluorescence, suggesting good permeability of the probe into the cells of *P. obducens*, without the need for additional cell pretreatment (Fig. 3a–i). The rRNA_ITS_Pob probe labeled with the Alexafluor 350 (blue) dye generally gave a less intense signal than the probe labeled with Alexafluor 594 (red), but still provided specific labeling of *P. obducens* sporangiophores and sporangia (Fig. 3g–i).

**Fig. 4:**
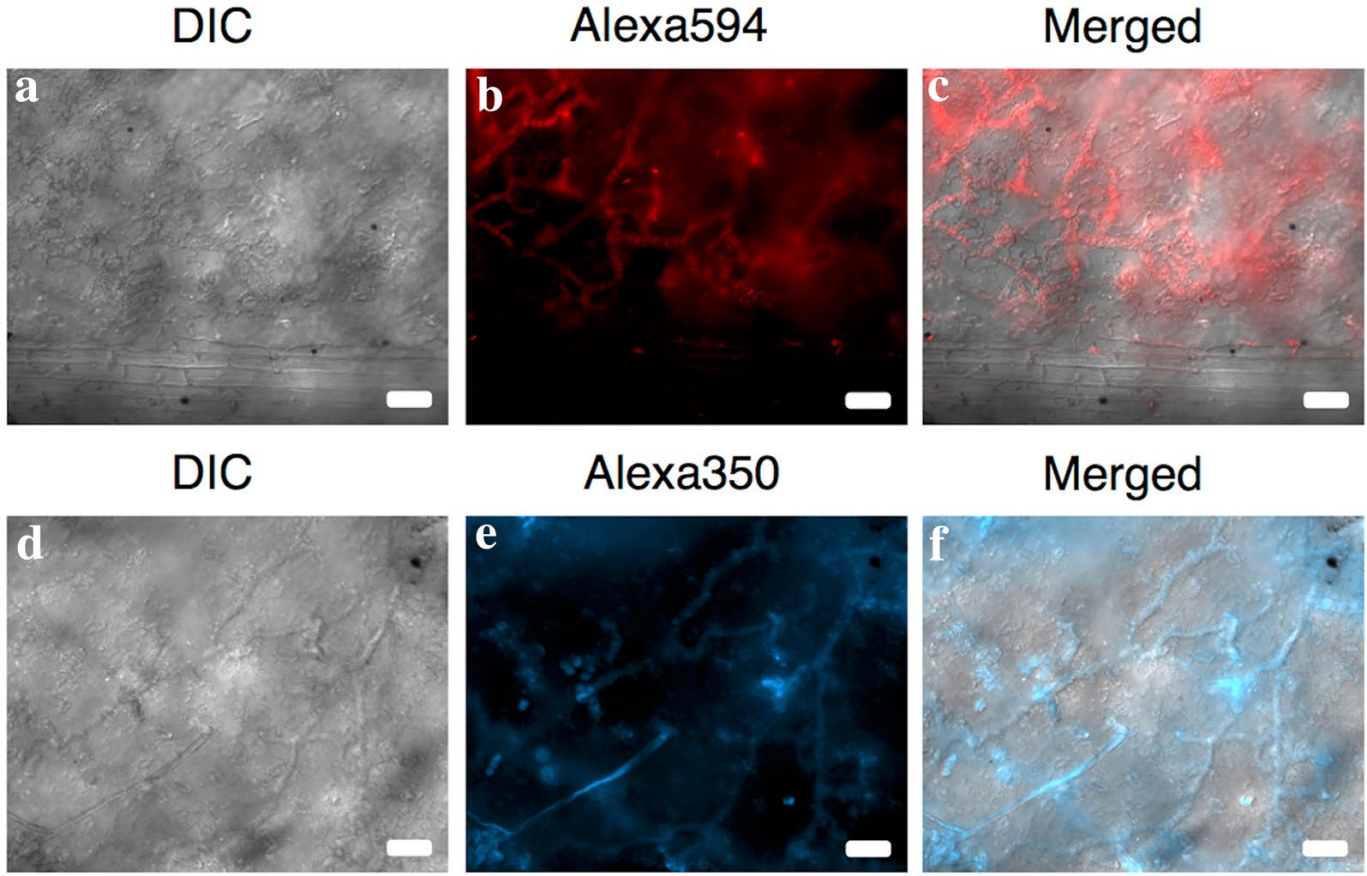
*Plasmopara obducens* colonizing *Impatiens walleriana* tissues as visualized on the abaxial leaf surface using probes labeled with Alexa595 fluorophore (red, **a**–**c**) or Alexa350 fluorophore (blue, **d**–**f**) fluorophores. Scale bar: **a**–**f** = 50 µm

**Fig. 5:**
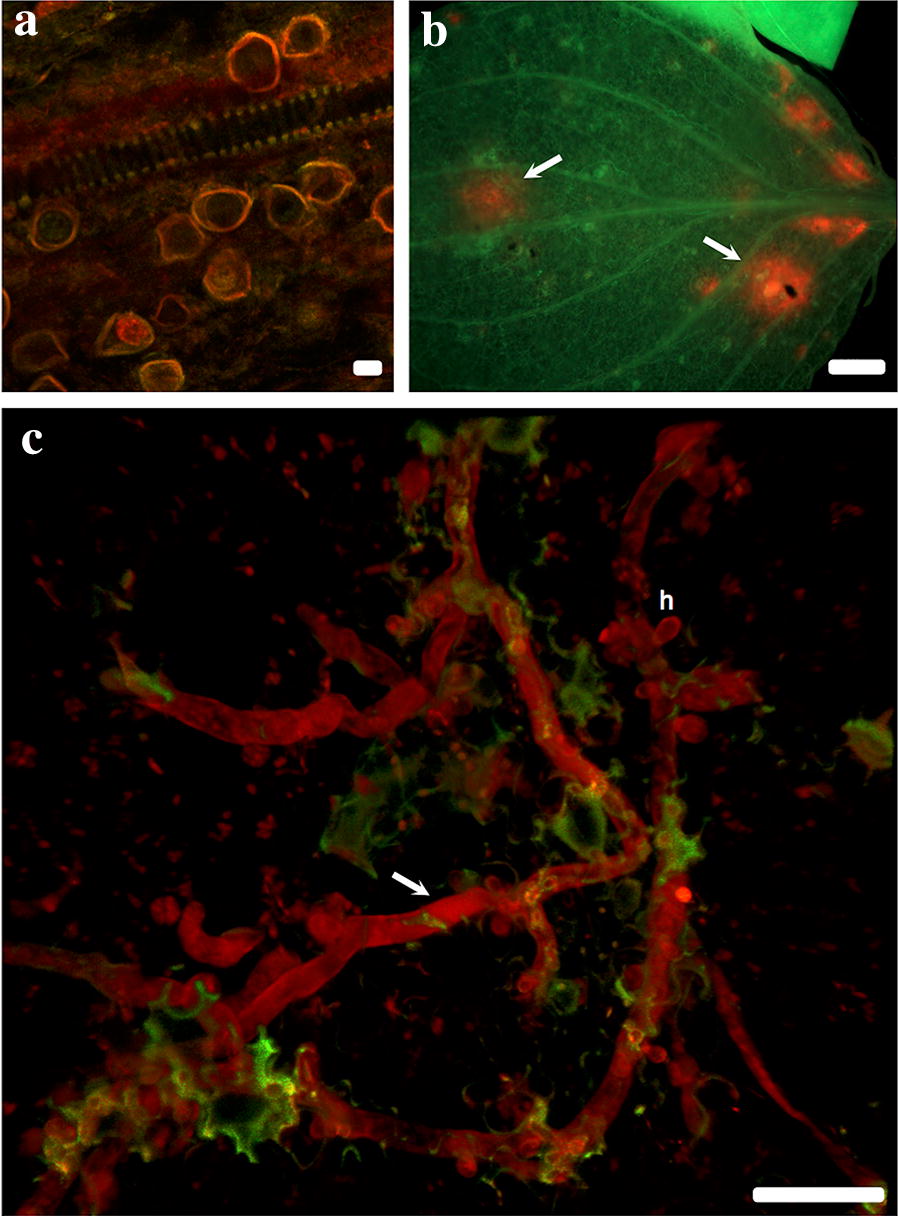
*Plasmopara obducens* oospores and mycelia colonizing *Impatiens walleriana* tissues as visualized using probe labeled with Alexa595 fluorophore. **a** Oogonia and oospores within stem tissue; **b** adaxial leaf surface displaying localized areas of infection indicated by arrows; **c** two-dimensional image of mycelia (arrow) in the leaf mesophyll (h = haustoria). Scale bar: **a** = 20 µm; **b** = 2 mm; **c** = 50 µm

Naturally infected *I. walleriana* leaves that were cleared previous to the FISH treatment produced fluorescence signal that corresponded to *P. obducens* hyphae growing within the palisade and spongy mesophyll (Figs. 4a–f, 5c). Fluorescence appeared especially intense on some areas of the *I. walleriana* leaves (Fig. 5b), mostly due to accumulated overlaying invading hyphae, which are common beneath the pathogen penetration sites and the presence of haustoria in mesophyll cells [34]. Based on these results, it appears that the probe has the ability to penetrate several layers of *I. walleriana* cells. This would allow for the application of this assay on a variety of *I. walleriana* plant tissues, without the need of histological sectioning. Mycelia was not observed within vascular tissue (Fig. 4a–c), however mature oospores within the stem cortex were detected, with the fluorescence localized in the area between the wall of the oospore and the oogonium (Fig. 5a).

The FISH probe developed in this study targets transcribed target rRNA, however its successful application relies on the presence of intact RNA molecules in the sample. The rRNA content of microorganisms has been found to be correlated the active growth and it is often used as an indicator of ongoing cellular activity [35]. The FISH method applied to a single dried herbarium specimen of *I. walleriana* infected with *P. obducens* failed to emit detectable fluorescence (data not shown). It is likely that fresh samples previously fixed using paraformaldehyde or any other preservation methods are better suited for the application of this FISH method.

## Conclusion

A novel probe was developed for FISH-based visualization of *P. obducens* on *I. walleriana*. FISH is a reliable tool to detect and localize a wide variety of microorganisms in different growing stages and from different sources. This is the first time this technique has been successfully applied for the visualization of plant pathogens in the Peronosporaceae that cause downy mildew disease. This technique is anticipated to be valuable in the early detection of infected *I. walleriana* plant material before the appearance of typical symptoms or the presence of pathogen structures on the exterior of the plant host, which are both indicative of advanced stages of disease development. Relative to the time required for manifestation of disease symptoms or for signs of the pathogen to become apparent—especially when fungicide products are used to suppress pathogen activity in the plant—FISH assays can be performed in about 2 days. FISH techniques have also been successfully used to elucidate host–pathogen interactions, which are essential for understanding the often-cryptic early stages of infection, the mechanisms of pathogen’s life cycle, virulence and host resistance [36, 37]. The general and molecular pathogenesis of the IDM disease system is poorly understood, yet FISH techniques and the probe developed in this study could be used to study the early phase of infection and subsequent colonization. The determination of key points in the disease progression that have an effect in the success of disease management practices, and the overall improved understanding of IDM are essential to reduce the effect of this devastating disease.
